# STK31/TDRD8, a Germ Cell-Specific Factor, Is Dispensable for Reproduction in Mice

**DOI:** 10.1371/journal.pone.0089471

**Published:** 2014-02-19

**Authors:** Jian Zhou, N. Adrian Leu, Sigrid Eckardt, K. John McLaughlin, P. Jeremy Wang

**Affiliations:** 1 Department of Animal Biology, University of Pennsylvania School of Veterinary Medicine, Philadelphia, Pennsylvania, United States of America; 2 Research Institute at Nationwide Children's Hospital, Columbus, Ohio, United States of America; University of Connecticut, United States of America

## Abstract

Tudor domain containing (Tdrd) proteins that are expressed in germ cells are divided into two groups. One group, consisting of TDRD1, TDRKH, TDRD9 and TDRD12, function in piRNA biogenesis and retrotransposon silencing, while the other group including RNF17/TDRD4 and TDRD5-7 are required for spermiogenesis. These Tdrd proteins play distinct roles during male germ cell development. Here, we report the characterization of STK31/TDRD8 in mice. STK31 contains a tudor domain and a serine/threonine kinase domain. We find that STK31 is a cytoplasmic protein in germ cells. STK31 is expressed in embryonic gonocytes of both sexes and postnatal spermatocytes and round spermatids in males. Disruption of the tudor domain and kinase domain of STK31 respectively does not affect fertility in mice. Our data suggest that the function of STK31 may be redundant with other Tdrd proteins in germ cell development.

## Introduction

The tudor domain was originally identified as a conserved structural motif of approximately 60 amino acids in *Drosophila* Tudor protein, which is required for assembly of polar granules in germ plasm [1]–[3]. Tudor domains mediate protein-protein interactions by recognizing and binding the methylated arginines or lysines of target substrates to assemble macromolecular complex or granules at discrete cellular compartments [4], [5]. The tudor proteins play important roles in many processes during development, such as genome stability, cell division and gametogenesis. Moreover, they are involved in many processes of RNA metabolism, including RNA splicing, miRNA/siRNA pathway, and piRNA pathway [6].

The piRNAs are a class of 26–31-nt germline-specific RNAs that are bound to members of the Piwi subfamily of Argonaute proteins. The function of piRNAs is associated with the silencing of transposons to protect the genome integrity during germ cell development [7], [8]. In mice, there are three members of Piwi proteins, MILI (PIWIL2), MIWI (PIWIL1) and MIWI2 (PIWIL4). These Piwi proteins harbor multiple methylated arginine sites at conserved positions in N termini [9]. Tudor domain containing (Tdrd) proteins recognize the methylated arginines and bind Piwi proteins to function in piRNA biogenesis and spermiogenesis. TDRD1 interacts with MILI and participates in the primary piRNA biogenesis in intermitochondrial cements [10], [11], while TDRD9 interacts with MIWI2 to function in secondary piRNA biogenesis in processing bodies [12]. TDRKH interacts with MIWI/MIWI2 and is involved in primary piRNA maturation [13]. TDRD12 interacts with MILI and is required for secondary piRNA biogenesis [14]. RNF17/TDRD4, TDRD6 and TDRD7 interact with MIWI and are essential for spermiogenesis [15]–[17]. TDRD5 is a component of intermitochondrial cement and chromatoid body and is required for repression of LINE1 transposons and spermiogenesis [18]. Therefore, Tdrd proteins are critical for male germ cell development.

*Stk31* was first identified as a germ cell-specific gene in a cDNA subtraction screen [19]. STK31 has been reported to interact with MIWI, suggesting a role in spermatogenesis [20]. To characterize the function of STK31, we made two lines of mutants by disrupting its tudor domain and kinase domain respectively. However, reproduction was not affected in either mutant, suggesting functional redundancy among Tdrd proteins.

## Results

### Specific expression of STK31 in germ cells

**Figure 1:**
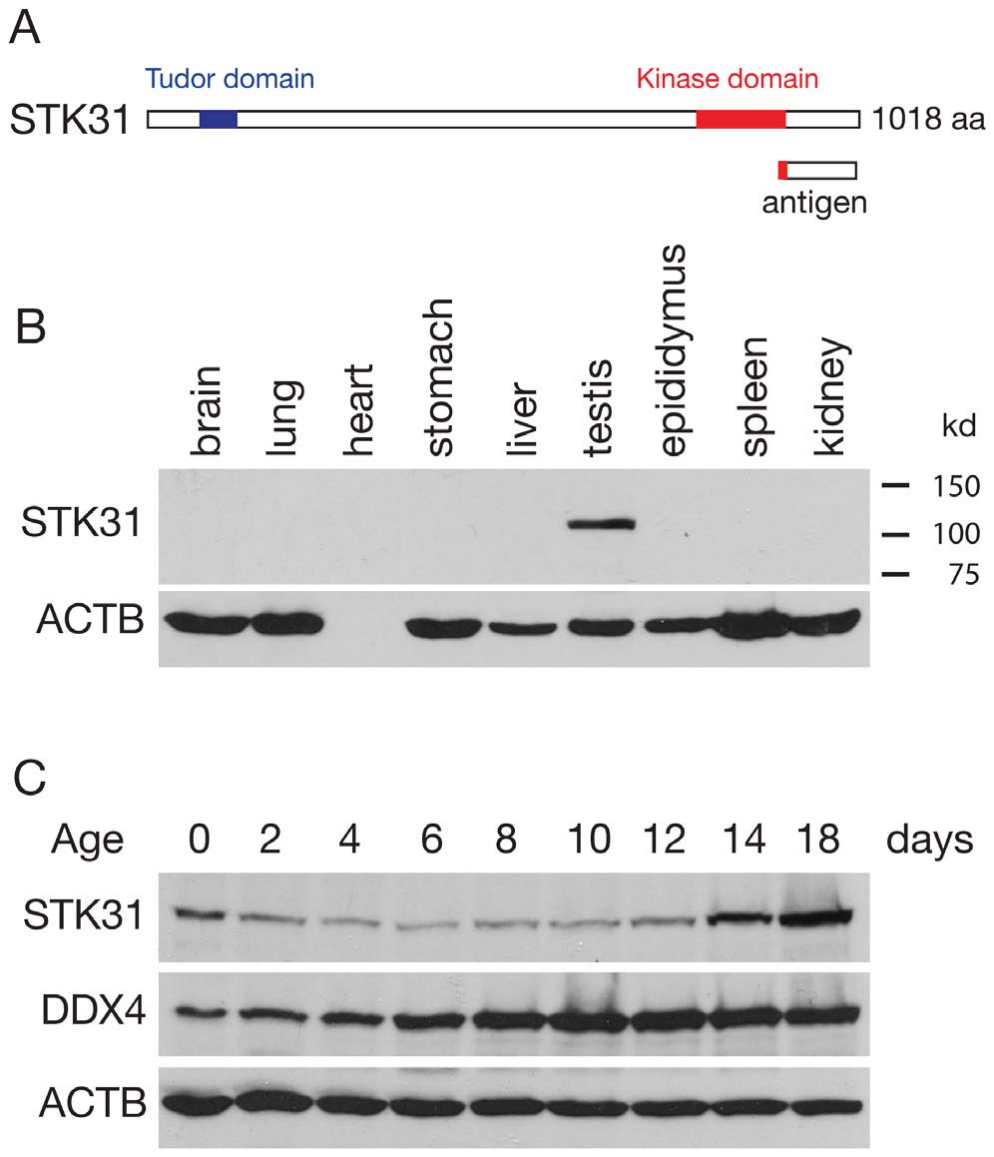
Tissue-specific and temporal expression of STK31. (A) Diagram of predicted tudor and kinase domains in mouse STK31 protein. The C-terminal (919–1018aa) fragment was used as antigen for antibody production. (B) Western blot analysis of STK31 in adult mouse tissues. Anti-STK31 antibody GP79 was used. (C) Western blot analysis of STK31 and DDX4 in testes from mice of different postnatal ages. DDX4 is germ cell-specific and is expressed from prespermatogonia to round spermatids in testes. Note that DDX4 expression increases with the development of postnatal testes and reaches the maximum around postnatal day 12. ACTB served as a ubiquitous control.

To examine the expression and localization of STK31, we generated antibodies against a mouse STK31 recombinant fusion protein (Fig. 1A). Western blot analysis showed that STK31 was specifically expressed in the testis in adult mice (Fig. 1B). We next examined the developmental expression pattern of STK31 in juvenile testes. STK31 was highly expressed in newborn testes, which contain gonocytes. Subsequently, STK31 abundance was decreased through postnatal day 8, when the only germ cells in the testis are spermatogonia. The low level of STK31 expression persisted in postnatal day 10 and 12 testes, in which leptotene and zygotene spermatocytes are present. The abundance of STK31 protein was significantly increased at day 14, when pachytene spermatocytes first appear (Fig. 1C). These data suggest that the STK31 expression was high in gonocytes and pachytene spermatocytes, but was low in spermatogonia.

**Figure 2:**
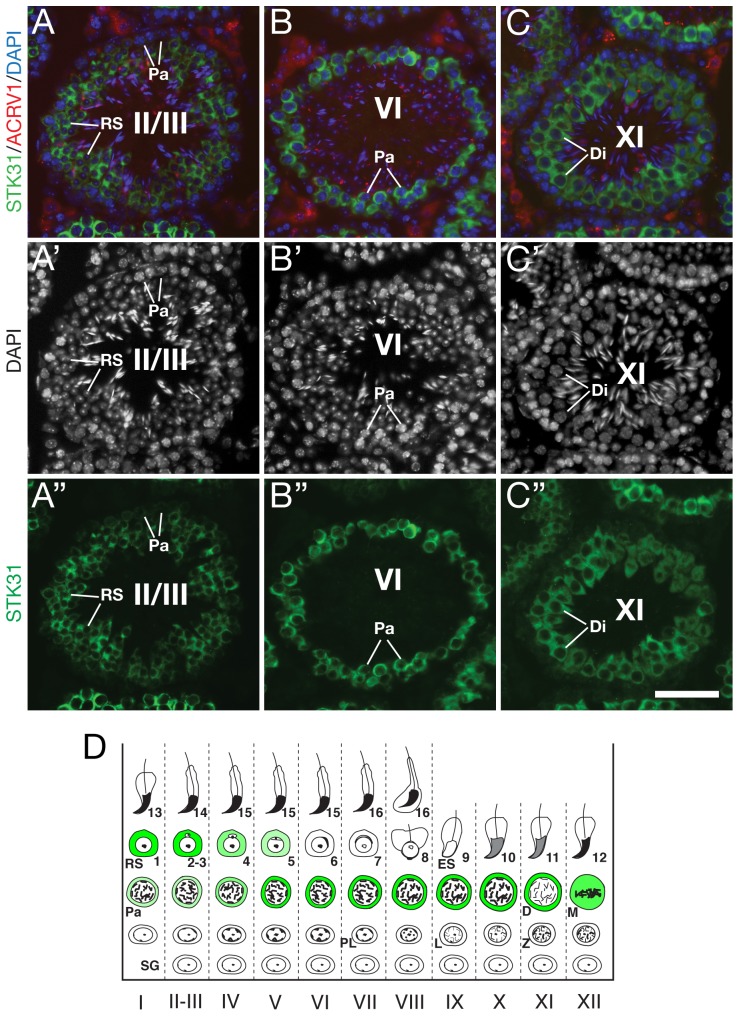
Expression pattern and localization of STK31 in adult testes. (A-C) Expression of STK31 in representative seminiferous tubules. Testis sections from adult mice were immunostained with anti-STK31 antibody UP2169 (green) and anti-ACRV1 antibody (red). Nuclei were stained with DAPI. The shape of acrosome showed by ACRV1 and the morphology of nuclei were used to determine the stages of seminiferous tubules. (D) Diagram of STK31 protein (green) localization during spermatogenesis. Stages (I-XII) of spermatogenesis are indicated below. Abbreviations: SG, spermatogonia; PL, preleptotene spermatocytes; L, leptotene spermatocytes; Z, zygotene spermatocytes; Pa, pachytene spermatocytes; RS, round spermatids; ES, elongating/elongated spermatids. Scale bar, 50 µm.

We next determined the spatiotemporal distribution of STK31 by immunostaining of adult testis sections. ACRV1 is a marker of acrosome [21]. To determine the stages of the STK31 expression, we performed double immunostaining of testis sections with anti-STK31 and anti-ACRV1 antibodies (Fig. 2). STK31 was expressed at a very low level in spermatogonia and prepachytene spermatocytes. STK31 was highly expressed in pachytene spermatocytes through round spermatids up to step 5. STK31 was not detectable in spermatids of step 6 and beyond. As expected, STK31 was not detected in epididymal sperm by western blot analysis (Fig. S1).

We then examined the expression of STK31 in embryonic germ cells. Immunofluorescence analysis revealed that STK31 expression in the testis started at E14.5 and significantly increased at E17.5 (Fig. S2A, B). These observations are consistent with human STK31 expression in gonocytes [22]. The *Stk31* expression in fetal mouse ovaries was reported by Olesen *et al* [23]. They performed microarray analysis to compare RNA profile of E13.5 ovaries with E11.5 ovaries and found that *Stk31* mRNA level was significantly increased. To determine the subcellular localization of STK31 in the ovary, we performed immunostaining of embryonic and postnatal ovaries. STK31 protein was localized in the cytoplasm of oocytes in E15.5 and E17.5 ovaries (Fig. S2C, D). MSY2 is a marker of oocytes in the ovary [24]. At postnatal day 10, STK31 was highly expressed in primordial follicles (Fig. S3). MSY2 expression was higher in growing follicles than in primordial follicles. In contrast, STK31 level was decreased in growing follicles compared with primordial follicles.

### STK31 localization overlaps with intermitochondrial cements and processing bodies

**Figure 3:**
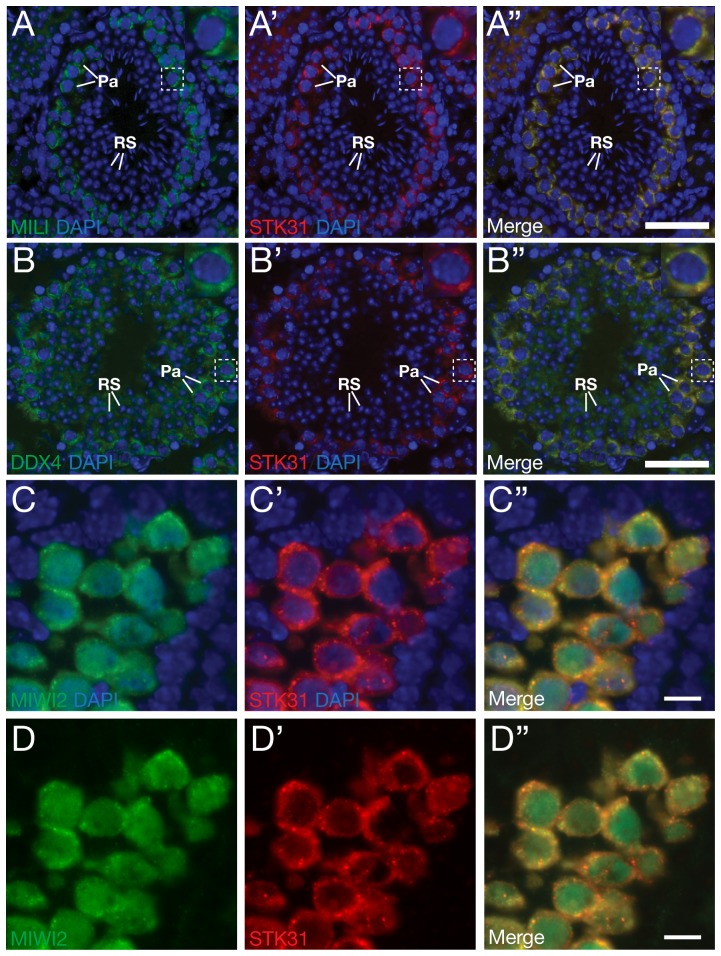
Co-localization of STK31 with MILI, DDX4 and MIWI2 in male germ cells. (A, B) Testis sections from adult mice were immunostained with anti-STK31 antibody GP79 (red) and anti-MILI antibody (green) or anti-DDX4 antibody (green). Nuclei were stained with DAPI. (C) Testis sections from E17.5 embryos were immunostained with anti-STK31 antibody GP79 (red) and anti-MIWI2 antibody (green). Pa, pachytene spermatocytes; RS, round spermatids. (D) The same images as in panel C without the DAPI channel. Scale bar, 50 µm (A, B), 10 µm (C, D).

The protein components of the piRNA pathway localize to cytoplasmic nuage in the germ cells, such as intermitochondrial cement (IMC) and processing body (P-body). For instance, MILI, TDRD1 and DDX4 localize to IMC in pro-spermatogonia and pachytene spermatocytes, while MIWI2 and TDRD9 are components of the P-body [10]–[12]. To exam potential co-localization of STK31 with the piRNA pathway components, we performed co-immunostaining analysis of STK31 with MILI, DDX4 and MIWI2. In spermatocytes, STK31 colocalized with MILI and DDX4 (Fig. 3A, B). In pro-spermatogonia, STK31 granules partially overlapped with cytoplasmic MIWI2 foci (Fig. 3C) but not with nuclear MIWI2 (Fig. 3D). These colocalization results suggest that STK31 may function in the piRNA pathway.

### Deletion of STK31 tudor domain in mice

**Figure 4:**
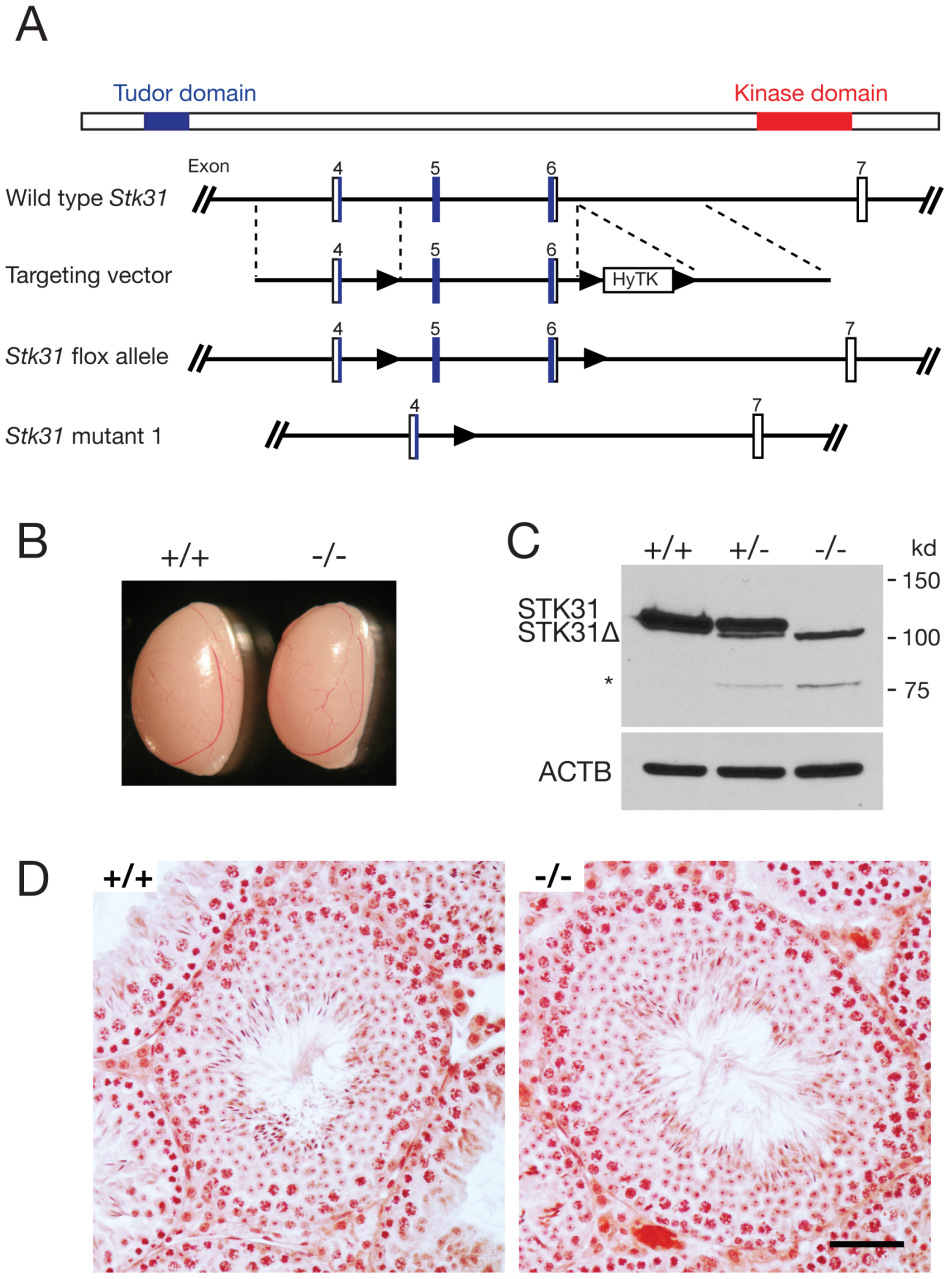
Tudor domain of STK31 is not required for spermatogenesis. (A) Targeted disruption of the tudor domain in the *Stk31* gene. Mouse *Stk31* gene (encoding a protein of 1018 aa) has 24 exons over a 74-kb genomic region on Chr. 6. The targeting strategy is to flox exon 5 and 6 (encoding 84–161 aa). The floxed region was removed by crossing with *ActB*-Cre mice. (B) Testis size of 8-wk-old wild type and mutant mice. (C) Western blot analysis of 8-wk-old wild type, *Stk31* ^+/−^ and *Stk31* ^−/−^ testes. The mutant protein (Stk31**Δ**) was less abundant. The small band (*) resulted possibly from degradation of the mutant protein or alternative splicing of the mutant allele. Anti-STK31 antibody GP79 was used. (D) Histological analysis of 8-wk-old wild type and mutant testes. Scale bar, 50 µm.

The *Stk31* gene encodes a protein of 1018 amino acids, containing a tudor domain close to its N terminus and a kinase domain at the C terminus (Fig. 4A). The conservative estimate of tudor domain is 81–135 amino acids (SMART) or 28–147 amino acids (PFAM). To specifically delete the tudor domain, we generated a conditional mutant allele (*Stk31* ^fl^) with one loxP site in intron 4 and one in intron 6 (Fig. 4A). Exons 5 and 6 encode aa 84–161. The mutant transcript was expected to maintain its reading frame. *Stk31* ^fl^ mice were crossed with *Actb*-Cre to delete exons 5 and 6. *Actb*-Cre is ubiquitously expressed [25]. The tudor deletion male and female mice appeared to be healthy and fertile. The size of mutant testes was comparable to that of controls (Fig. 4B). Western blot analysis with our STK31 antibody GP79 showed that the mutant protein was present and appeared to be less abundant (Fig. 4C). Histology of mutant testis appeared to be normal (Fig. 4D). These mice were generated on a mixed genetic background. After backcrossing to the C57BL/6J strain for six generations, mutant male mice were still fertile with normal testis weight and normal sperm count (Table 1). In summary, the tudor domain of STK31 is dispensable for spermatogenesis in mice.

**Table 1:** Testis weight and sperm production in *Stk31* mutant mice.

	Tudor domain deletion^a^			Kinase domain deletion^a^		
	+/+ or +/– (n = 8)	–/– (n = 8)	P-value^b^	+/+ or +/– (n = 7)	–/– (n = 7)	P-value^b^
Body weight (g)	23.0±1.8	23.8±1.5	0.36	23.4±1.0	23.5±1.1	0.9
Testis weight (mg)	170.8±19.8	179.5±13.0	0.32	169.9 ±15.2	173.2±10.9	0.65
Sperm/cauda (10^6^)	8.6±1.5	8.7±1.5	0.88	8.5±1.4	8.9±1.1	0.59

a Mice backcrossed to the C57BL/6J strain for six generations for tudor domain deletion mutant and ten generations for kinase domain deletion mutant were used at 8 weeks of age.

b By *Student*’s T-test.

### Disruption of STK31 kinase domain in mice

**Figure 5:**
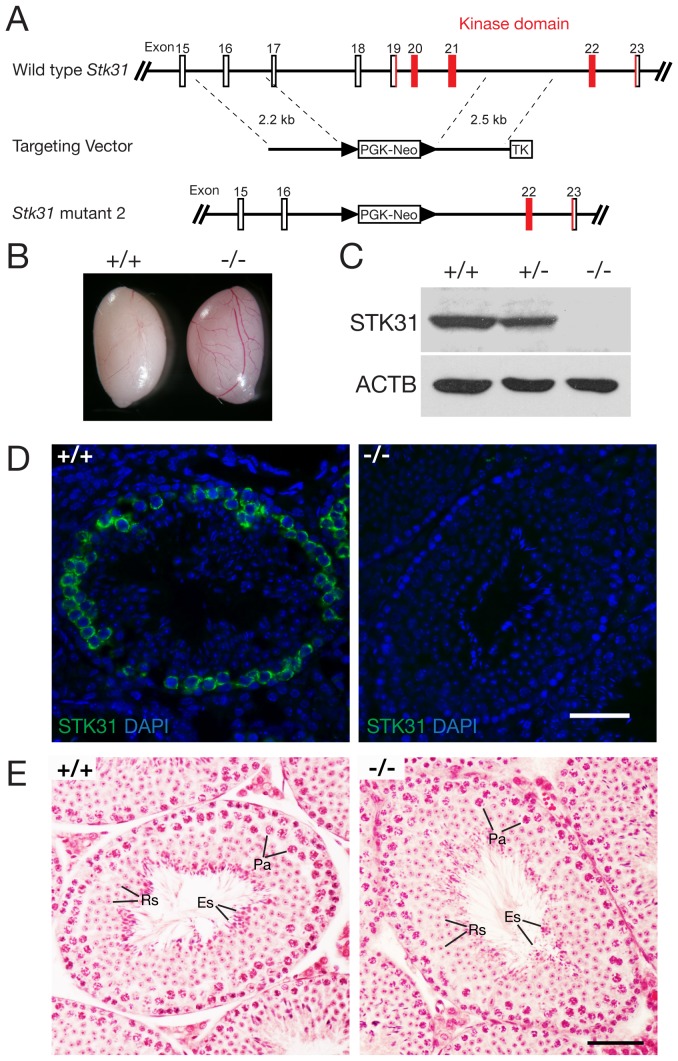
The kinase domain of STK31 is dispensable for spermatogenesis. (A) Targeted deletion of the kinase domain in the *Stk31* gene. The targeting strategy is to delete exons 17–21 (5.16 kb genomic region) by replacing it with the PGK-Neo selection cassette. (B) Comparable size of wild type and mutant testes from mice at the age of 8 weeks. (C) Western blot analysis of 8-wk-old wild type, *Stk31* ^+/−^, and *Stk31* ^−/−^ testes. The mutant protein was undetectable. Anti-STK31 antibody GP79 was used. (D) Immunofluorescence analysis of the STK31 protein in adult wild type and *Stk31* ^−/−^ testes. (E) Histological analysis of 8-wk-old wild type and *Stk31* ^−/−^ testes. Scale bar, 50 µm.

The kinase domain remained intact in the *Stk31* mouse mutant with the deletion of its tudor domain. To disrupt the kinase domain, we used the neo cassette to replace the DNA fragment harboring exons 17–21, removing residues 691–880 (Fig. 5A). The most conservative prediction of the kinase domain is 800–923 amino acids, starting in exon 19. Interbreeding of heterozygous mice yielded a normal Mendelian ratio of offspring (+/+, +/–, –/–: 57, 102, 59), suggesting lack of lethality in *Stk31*-deficient pups. Both *Stk31* ^−/−^ male and female mice appeared to be grossly healthy and fertile. The size of *Stk31* ^−/−^ testis was comparable to that of wild type littermates (Fig. 5B). RT-PCR and sequencing results confirmed that exon 22 is linked to exon 16 in the *Stk31* mutant mRNA from *Stk31* ^−/−^ testis. Exons 22-24 were still in frame and contained the antigen fragment for our anti-STK31 antibody GP79 (Fig. 1A). However, western blot analysis with antibody GP79 showed that STK31 protein was absent in the *Stk31* ^−/−^ testis (Fig. 5C). In addition, immunofluorescence analysis with antibody GP79 showed the absence of STK31 in the *Stk31* ^−/−^ testis (Fig. 5D). These results showed that this mutant lacking the STK31 kinase domain is null. Histology of *Stk31* ^−/−^ testis appeared to be normal (Fig. 5E). These mice were generated on a mixed genetic background. After backcrossing to the C57BL/6J strain for ten generations, *Stk31* ^−/−^ male mice were still fertile with normal testis weight and normal sperm count (Table 1).

### STK31 is not a component of the DNA damage checkpoint in oocytes

Since STK31 is expressed in oocytes throughout oogenesis, we wondered whether it plays a role in the DNA damage checkpoint, which triggers apoptosis of oocytes upon DNA damage. To test this possibility, we treated young pups with γ-irradiation and analyzed the survival of oocytes by immunostaining with MSY2 antibody [24], [26], [27]. Both wild type and mutant ovaries lack primordial follicles, suggesting that the DNA damage checkpoint is intact (Fig. S4). This result suggests that STK31 did not function in DNA damage-induced apoptosis in ovaries.

## Discussion

Previous genetic studies have shown that Tdrd proteins play distinct roles in piRNA biogenesis and spermiogenesis. However, our present study demonstrates that STK31 is not essential for spermatogenesis. A recent study also showed that STK31 is dispensable for spermatogenesis [28]. Except STK31, six members of Tdrd proteins are expressed in pre-spermatogonia. In *Tdrkh*, *Tdrd9* and *Tdrd12* mutants, male germ cell development is arrested at the zygotene stage of meiosis [12]–[14]. In *Tdrd1*, *Tdrd5* and *Tdrd7* mutants, LINE1 expression is derepressed and the apoptotic cells increase, but at least part of germ cells go through meiosis and arrest at round spermatid stage [17], [18], [29]. TDRD1, TDRD5, TDRD6 and TDRD7 are components of chromatoid bodies in round spermatids and RNF17 forms distinct granules in spermatocytes. They are required for post-meiotic germ cell development [30], [31]. STK31 does not localize to chromatoid bodies or RNF17 granules. Recently, two studies reported that STK31 localizes to equatorial segment of acrosome in sperm [20], [32]. In contrast, we did not observe this signal in immunofluorescence assay. Our western blot analysis showed that STK31 was undetectable in epididymis and isolated epididymal sperm. Our antibody is highly specific. Therefore, the previous report on the localization of STK31 in sperm might result from non-specific cross-reaction of their antibodies [20], [32].

Several Tdrd proteins are expressed in mouse ovary. TDRD1 and TDRD9 are expressed in growing oocytes, but show different localization [12], [29]. TDRD1 only localizes to the cytoplasm with a granular appearance, while TDRD9 localizes to both cytoplasm and nucleus without any granule. TDRD5 is also expressed in the ovary, but its localization has not been reported [18]. STK31 is expressed in oocytes from embryonic stage to adult and localizes to cytoplasm without particular appearance. However, the female mutants of all Tdrd and Piwi factors are fertile. Recently, it has been hypothesized that MARF1 instead silences retrotransposons in mouse female germline [33], [34].

Protein phosphorylation and dephosphorylation have been illustrated in many signaling pathways, including the apoptotic pathway [35]. A recent study showed that TDRKH-deficient spermatocytes were eliminated through a non-apoptotic pathway, suggesting a role of Tdrd proteins in the cell death pathway [13]. STK31 is highly expressed in primordial follicle oocytes in ovaries. The primordial follicle oocytes are very sensitive to DNA damage. The γ-irradiation-induced DNA damage results in apoptosis of primordial follicle oocytes through activation of p63 [26]. STK31 deficiency does not rescue primordial follicle oocytes from apoptosis, suggesting that STK31 may not function in apoptotic pathway in oocytes.

A number of germline-specific genes are expressed in human cancers [36]. These factors are potential targets for cancer immunotherapy and are thus referred to as “cancer/testis antigens” [37]. Human *STK31* was reported to be expressed in gastrointestinal cancers, including esophageal, gastric, colonic and colorectal cancers [38], [39]. Knockdown of *STK31* in colonic cancer cells promotes cell differentiation and thus suppresses tumorigenicity [39]. In addition, the kinase domain in STK31 is required for its tumor-suppressing activity. Therefore, STK31 might be involved in tumorigenesis and is a potential target for immunotherapy in gastrointestinal cancers.

In summary, we described the expression and localization of STK31 in male and female germ cells. Our two mutants of *Stk31* showed that this gene was dispensable for spermatogenesis and oogenesis. To elucidate the redundant role of Tdrd proteins, double or triple knockout of *Stk31* with other Tdrd genes may be necessary. Furthermore, our *Stk31* mutant mice will be useful for genetically testing the tumor-suppressing activity of STK31 in gastrointestinal cancer mouse models.

## Materials and Methods

### Ethics statement

Mice were maintained and used according to standards approved by the Institutional Animal Care and Use Committee (IACUC) of the University of Pennsylvania. Full details of the study were approved in IACUC protocol # 804050. Tissues were collected after euthanasia. Mice were euthanized by CO_2_ inhalation and were monitored to assure that respiration has stopped and does not resume.

### Generation of antibodies

GST-mouse STK31 (amino acids 919–1018) was expressed in *E. coli* strain BL21 using the pGEX-4T-1 vector (Fig. 1A). After affinity purification with glutathione sepharose, the fusion protein was used to immunize rabbits and guinea pigs, resulting in anti-STK31 rabbit serum UP2169 and guinea pig serum GP79 (Cocalico Biologicals). The antibodies were purified by affinity immunoblotting and were diluted (1∶50) for western blot analysis.

### Targeted disruption of the *Stk31* gene

Mutant 1 (Fig. 4A): disruption of the tudor domain in the *Stk31* gene. Three DNA fragments (2.2 kb, 2.9 kb and 2.3 kb) were amplified by high-fidelity PCR using an *Stk31*-containing BAC clone (RP23-64D14) as template. The HyTK cassette was flanked by two loxP sites in the targeting construct. The V6.5 ES cells were electroporated with the linearized construct (*Cla*I) and selected in the presence of hygromycin (120 µg/ml, Roche). By screening 196 ES clones, we identified eight *Stk31* ^3lox^ clones. Two *Stk31* ^3lox^ ES cell lines were electroporated with the Cre-expressing pOG231 plasmid to remove the HyTK cassette. Two ES clones were injected into B6C3F1 (Taconic) blastocysts and the *Stk31* ^fl^ allele was transmitted through the germline in chimeric mice. *Stk31* ^fl^ mice were crossed with the ubiquitously expressing *Actb*-Cre to delete *Stk31* floxed region [25]. The *Stk31* mutant (479 bp) allele was assayed by PCR with primers AAGTCCAGCAATCAAAAGCC and GTAGCCTAGTATAATGGACTCTGG.

Mutant 2 (Fig. 5A): deletion of the kinase domain in the *Stk31* gene. Two DNA fragments (2.2 kb and 2.5 kb) were amplified using the same BAC clone as template. The Neo and HyTK cassettes were used for positive and negative selections respectively. The V6.5 ES cells were electroporated with the linearized construct (*Cla*I) and selected in the presence of G418 (350 µg/ml, Gibco) and ganciclovir (2 µM, Sigma). By screening 196 ES clones, we identified one *Stk31* targeted clone (2A9). The *Stk31* mutant allele was transmitted through the germline in chimeric mice. All offspring were genotyped by PCR with the primers GGGATACCAAGTGAGAGCTGTC and CCAAACCCTATGTCTTCCTACTC for the wild type allele (527 bp), TTCCTGAAAAGCAACATACTACAGTT and CCTACCGGTGGATGTGGAATGTGTG for the mutant allele (335 bp).

### Western blot

The tissues collected from mice at different ages were homogenized in the SDS-PAGE sample buffer and then were heated at 95°C for 10 minutes. Protein lysates were separated on 10% SDS-PAGE gel and electro-blotted on PVDF membranes. The primary antibodies used for western blot analysis were anti-STK31 GP79 (this study), anti-DDX4 (a gift from T. Noce) [40], anti-ACTB (Sigma-Aldrich), and anti-MNS1 UP2060 [41].

### Histological and immunofluorescence analyses

For histology, testes were fixed in Bouin’s solution overnight, processed, sectioned, and stained with hematoxylin and eosin. For immunofluorescence analysis, ovaries and testes were fixed in 4% PFA at 4°C for 3 hours and overnight respectively, dehydrated in 30% sucrose overnight, frozen in dry ice/95% ethanol, and sectioned at –20°C. The primary antibodies used for immunofluorescence analysis were anti-STK31 UP2169 and GP79 (this study), anti-ACRV1 (a gift from P. Reddi) [21], anti-DDX4 (a gift from T. Noce) [40], anti-MIWI2 (a gift from R. Pillai), anti-Mili (Abcam) and anti-MSY2 (a gift from R. Schultz) [24].

## Supporting Information

Figure S1 **The STK31 protein was undetectable in the sperm from cauda epididymis.** Sperm were collected from wild type cauda epididymis. Western blot analysis of testis and sperm was performed using anti-STK31 antibody GP79 and anti-MNS1 antibody UP2060. MNS1, a component of sperm flagella, served as a control.
(TIF)

Figure S2 **Expression of STK31 in embryonic testes and ovaries.** (A, B) Sections from embryonic testes were immunostained with anti-STK31 antibody GP79 (green). The STK31protein was detected in testis at E14.5 (A). Large granules of STK31 were observed in the cytoplasm of gonocytes at E17.5 (B). (C, D) STK31 expression in female germ cells. The STK31protein was detected in ovary at E15.5 (C). No STK31 granule was observed in oocytes at E17.5 (D). Scale bar, 50 µm.
(TIF)

Figure S3 **Dynamic expression and localization of STK31 in primordial and growing follicles.** Ovary sections from 10-day-old wild type (A) and kinase domain mutant (B) mice were immunostained with anti-STK31 antibody GP79 (green) and anti-MSY2 antibody (red). Nuclei were stained with DAPI. The abundance of STK31 protein was high in primordial follicles and low in growing follicles. STK31 protein was undetectable in the mutant ovary. PF, primordial follicle; GF, growing follicle. Scale bar, 50 µm.
(TIF)

Figure S4 **Loss of STK31 did not rescue primordial follicle oocytes from DNA damage-induced apoptosis.** Female pups (postnatal day 5) were untreated (A) or exposed to 0.45 Gy of γ-irradiation (B, C). Ovary sections from wild type and STK31 kinase domain mutant mice at postnatal day 10 were stained with anti-MSY2 antibody (green). PF, primordial follicle; GF, growing follicle. Scale bar, 50 µm.
(TIF)
