# Supplementary figures and images for: STK31/TDRD8, a Germ Cell-Specific Factor, Is Dispensable for Reproduction in Mice
Source: PLoS One. 2014 Feb 19;9(2):e89471. doi: 10.1371/journal.pone.0089471 (PMC3929691; doi:10.1371/journal.pone.0089471)

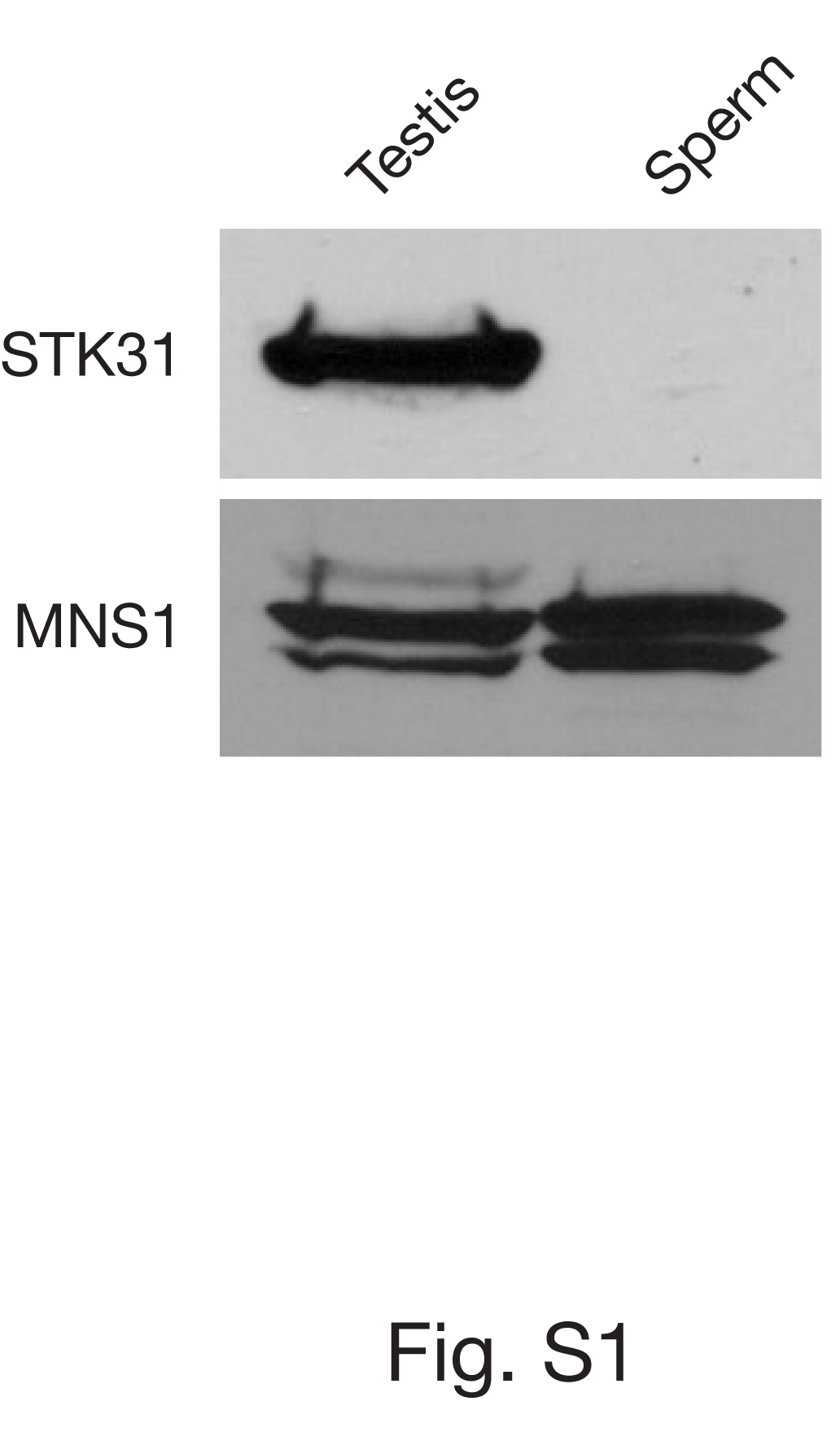

Supplement: Figure S1 — The STK31 protein was undetectable in the sperm from cauda epididymis. Sperm were collected from wild type cauda epididymis. Western blot analysis of testis and sperm was performed using anti-STK31 antibody GP79 and anti-MNS1 antibody UP2060. MNS1, a component of sperm flagella, served as a control. (TIF) [file pone.0089471.s001.tif]

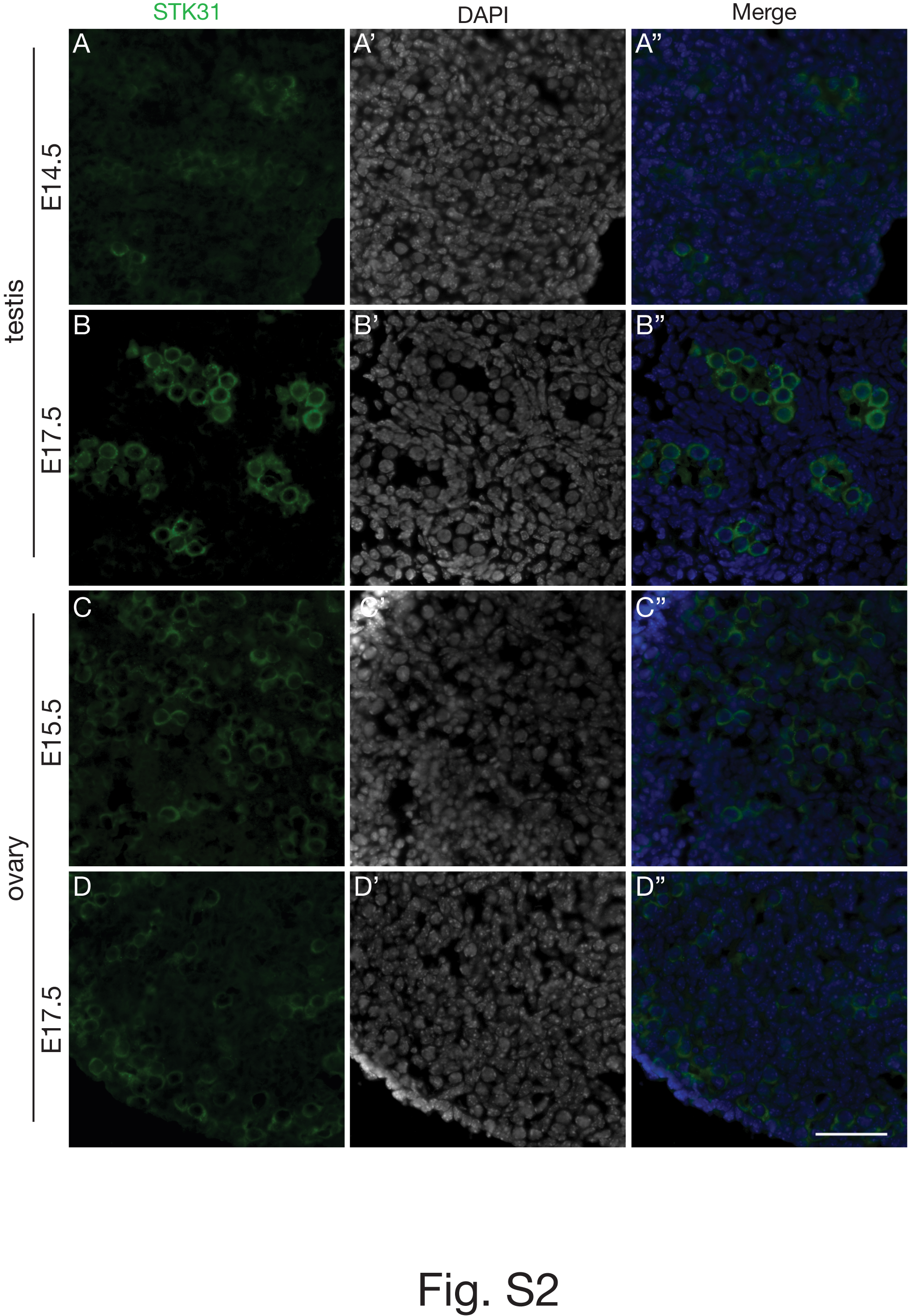

Supplement: Figure S2 — Expression of STK31 in embryonic testes and ovaries. (A, B) Sections from embryonic testes were immunostained with anti-STK31 antibody GP79 (green). The STK31protein was detected in testis at E14.5 (A). Large granules of STK31 were observed in the cytoplasm of gonocytes at E17.5 (B). (C, D) STK31 expression in female germ cells. The STK31protein was detected in ovary at E15.5 (C). No STK31 granule was observed in oocytes at E17.5 (D). Scale bar, 50 µm. (TIF) [file pone.0089471.s002.tif]

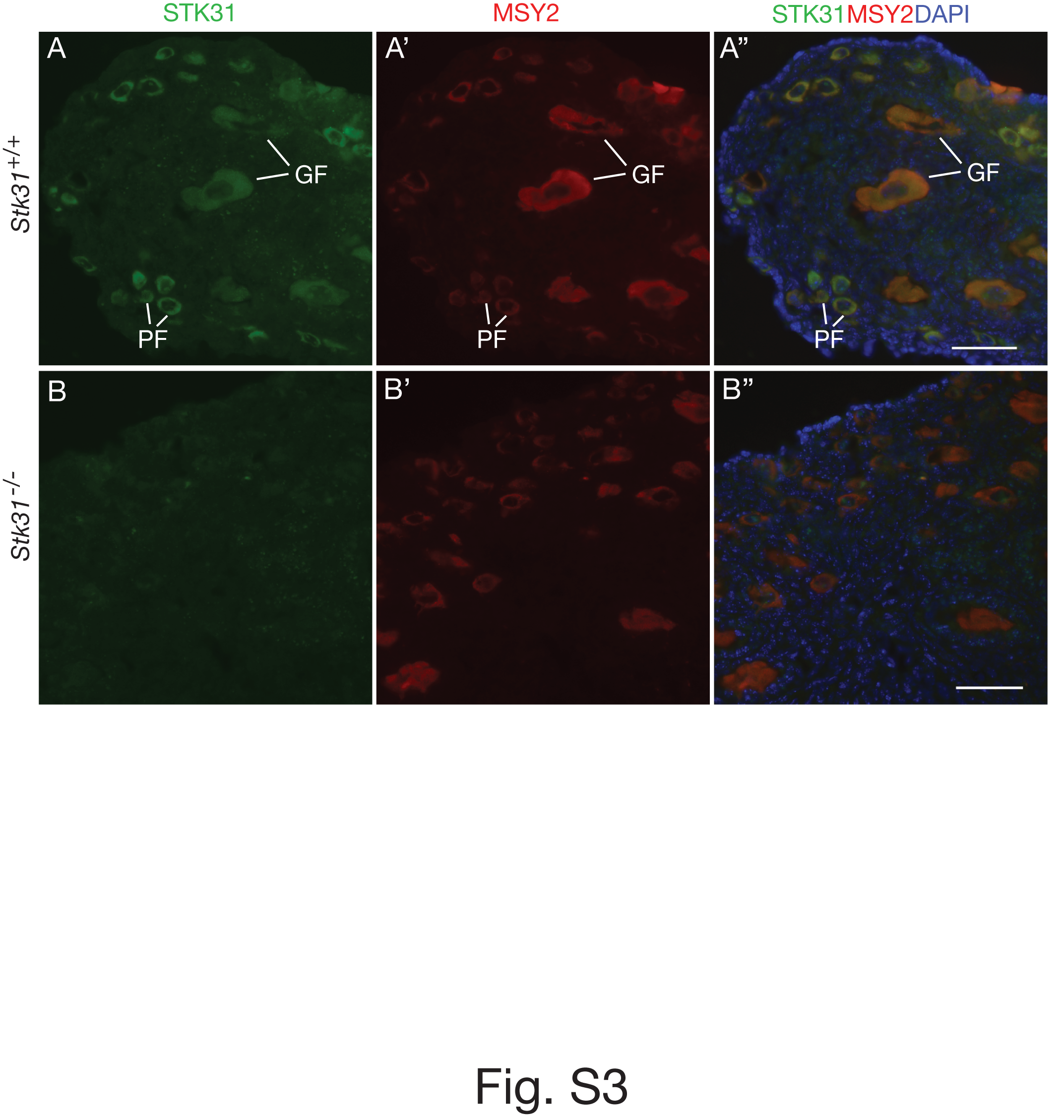

Supplement: Figure S3 — Dynamic expression and localization of STK31 in primordial and growing follicles. Ovary sections from 10-day-old wild type (A) and kinase domain mutant (B) mice were immunostained with anti-STK31 antibody GP79 (green) and anti-MSY2 antibody (red). Nuclei were stained with DAPI. The abundance of STK31 protein was high in primordial follicles and low in growing follicles. STK31 protein was undetectable in the mutant ovary. PF, primordial follicle; GF, growing follicle. Scale bar, 50 µm. (TIF) [file pone.0089471.s003.tif]

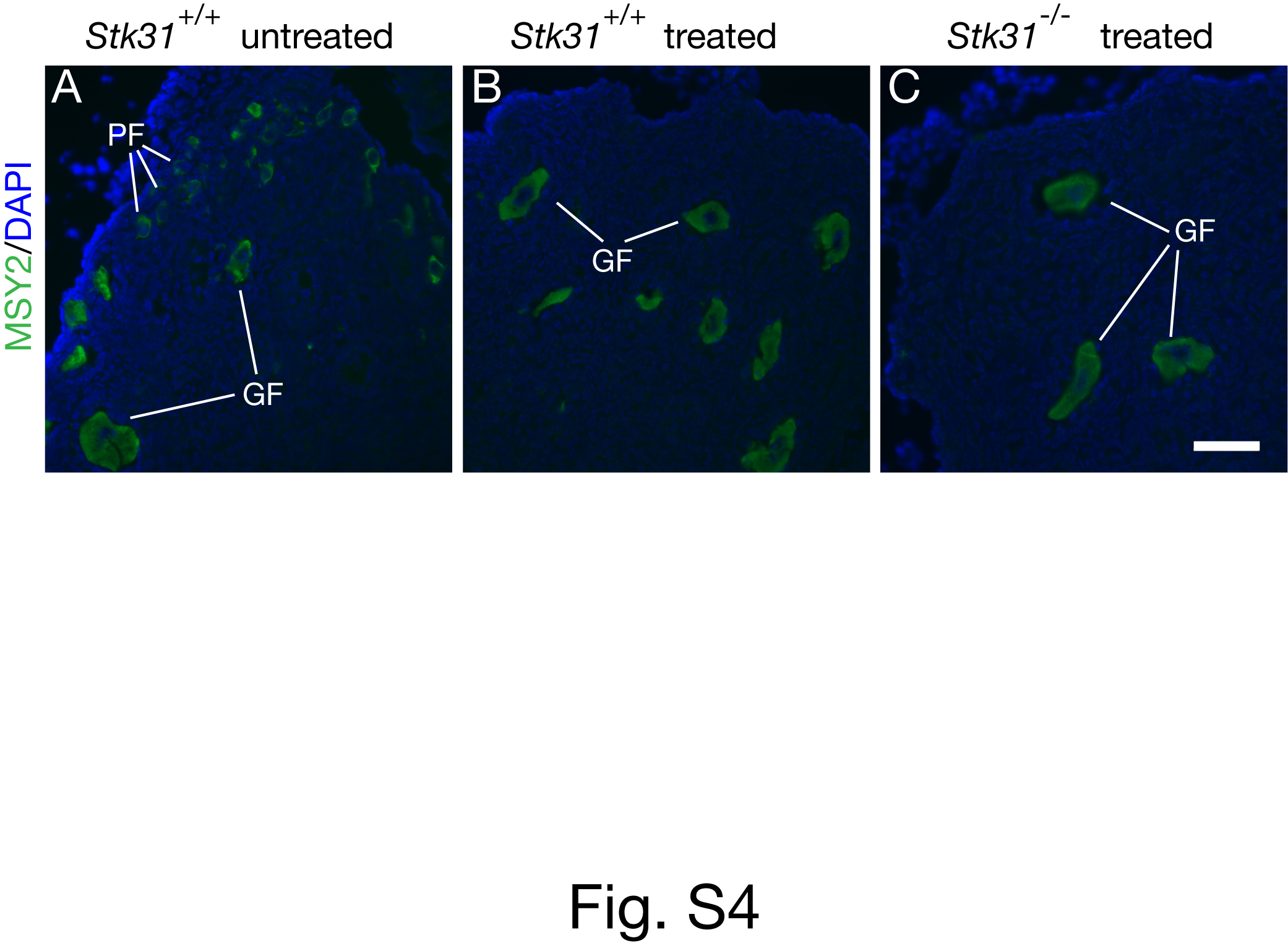

Supplement: Figure S4 — Loss of STK31 did not rescue primordial follicle oocytes from DNA damage-induced apoptosis. Female pups (postnatal day 5) were untreated (A) or exposed to 0.45 Gy of γ-irradiation (B, C). Ovary sections from wild type and STK31 kinase domain mutant mice at postnatal day 10 were stained with anti-MSY2 antibody (green). PF, primordial follicle; GF, growing follicle. Scale bar, 50 µm. (TIF) [file pone.0089471.s004.tif]
